# Supplementary material for: Splice-Junction-Based Mapping of Alternative Isoforms in the Human Proteome
Source: Cell Rep. Author manuscript; Available in PMC 2020 Jan 15. (PMC6961840; doi:10.1016/j.celrep.2019.11.026)
Supplement: 2 [file NIHMS1546469-supplement-2.zip › DF1/PXD006675/AorticValve/AorticValve_6_MACF1_GSNSEIDVDSLNLCLQQYETLQK.pdf]

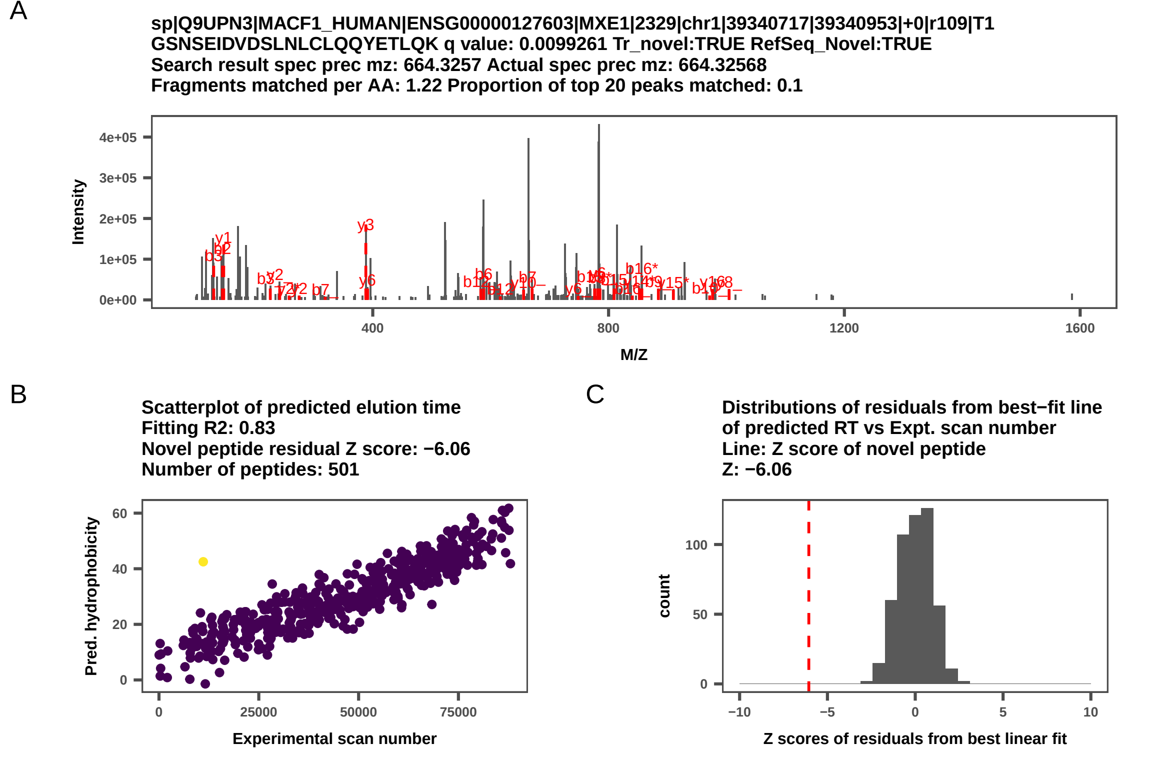
A
sp|Q9UPN3|MACF1_HUMAN|ENSG00000127603|MXE1|2329|chr1|39340717|39340953|+0|r109|T1
GSNSEIDVDSLNLCLQQYETLQK q value: 0.0099261 Tr_novel:TRUE RefSeq_Novel:TRUE
Search result spec prec mz: 664.3257 Actual spec prec mz: 664.32568
Fragments matched per AA: 1.22 Proportion of top 20 peaks matched: 0.1
Intensity
4e+05
3e+05
2e+05
1e+05
0e+00
400
800
1200
1600
M/Z
B
Scatterplot of predicted elution time
Fitting R2: 0.83
Novel peptide residual Z score: −6.06
Number of peptides: 501
Pred. hydrophobicity
60
40
20
0
0
25000
50000
75000
Experimental scan number
C
Distributions of residuals from best−fit line
of predicted RT vs Expt. scan number
Line: Z score of novel peptide
Z: −6.06
count
100
50
0
−10
−5
0
5
10
Z scores of residuals from best linear fit
